# MiR-449a promotes breast cancer progression by targeting CRIP2

**DOI:** 10.18632/oncotarget.7753

**Published:** 2016-02-26

**Authors:** Wei Shi, Jeff Bruce, Matthew Lee, Shijun Yue, Matthew Rowe, Melania Pintilie, Ryunosuke Kogo, Pierre-Antoine Bissey, Anthony Fyles, Kenneth W. Yip, Fei-Fei Liu

**Affiliations:** ^1^ Princess Margaret Cancer Centre, University Health Network, Toronto, Canada; ^2^ Division of Biostatistics, Princess Margaret Cancer Centre, University Health Network, Toronto, Canada; ^3^ Department of Radiation Oncology, Princess Margaret Hospital, Toronto, Canada; ^4^ Department of Radiation Oncology, University of Toronto, Toronto, Canada; ^5^ Department of Medical Biophysics, University of Toronto, Toronto, Canada

**Keywords:** breast cancer, miR-449a, metastasis, CRIP2, prognostic marker

## Abstract

The identification of prognostic biomarkers and their underlying mechanisms of action remain of great interest in breast cancer biology. Using global miRNA profiling of 71 lymph node-negative invasive ductal breast cancers and 5 normal mammary epithelial tissues, we identified miR-449a to be highly overexpressed in the malignant breast tissue. Its expression was significantly associated with increased incidence of patient relapse, decreased overall survival, and decreased disease-free survival. *In vitro*, miR-449a promoted breast cancer cell proliferation, clonogenic survival, migration, and invasion. By utilizing a tri-modal *in silico* approach for target identification, Cysteine-Rich Protein 2 (CRIP2; a transcription factor) was identified as a direct target of miR-449a, corroborated using qRT-PCR, Western blot, and luciferase reporter assays. MDA-MB-231 cells stably transfected with CRIP2 demonstrated a significant reduction in cell viability, migration, and invasion, as well as decreased tumor growth and angiogenesis in mouse xenograft models. Our data revealed that overexpression of miR-449a suppresses CRIP2, which then affects the tumor vasculature, likely *via* NF-κB/p65 complex-mediated transcription of VEGF. These finding define an oncogenic function of miR-449a in human breast cancer, and highlight the importance of this pathway in driving aggressive behaviour.

## STATEMENT OF TRANSLATIONAL RELEVANCE

The identification and characterization of prognostic biomarkers holds significant clinical utility for the most common subtype of breast cancer, invasive ductal breast carcinoma. In this manuscript, a novel prognostic microRNA, miR-449a, was identified to be overexpressed in lymph node-negative invasive ductal breast cancer using global miRNA profiling of 71 primary tumors and 5 normal mammary epithelial tissues. miR-449a was implicated functionally in breast cancer pathogenesis, suppressing Cysteine-Rich Protein 2 (CRIP2) and altering cell viability, migration, invasion, *in vivo* tumor growth, and angiogenesis, thereby driving malignant phenotypes in these aggressive tumors. Importantly, when paired with comprehensive patient follow-up data, expression of miR-449a was significantly correlated with higher risk of patient relapse, and decreased overall and disease-free survival rates, thus highlighting the possible applications for this newly characterized potential biomarker.

## INTRODUCTION

MicroRNAs (miRNAs; miRs) are a class of 17-22 nt non-coding RNAs that suppress other RNAs, and are master regulators of diverse cellular processes [1]. The abnormal expression of various miRNAs has been clearly demonstrated in a wide range of human malignancies, including leukemias and lymphomas [2], as well as colorectal [3], hepatocellular [4], head and neck [5], and breast cancers [6]. These irregular expression profiles have been phenotypically linked to increased proliferation, migration, and invasion [2, 7], with associated chemo- and radio-resistance [8]. In breast cancer, global miRNA expression profiling studies have identified significant alterations in a number of oncogenic and tumor-suppressive miRNAs, including miR-221, miR-21, miR-125, and miR-301 [9-11].

Breast cancer is currently the most frequently diagnosed cancer in women, and represents the second most common cause of cancer-related deaths in women in the developing world [12]. Since 2008, breast cancer incidence has increased by more than 20%, and mortality has increased by 14% [13]. The predominant histological subtype of breast cancer is invasive ductal carcinoma (IDC), which accounts for 80% of all cases in North America [14]. Identifying a prognostic biomarker capable of stratifying patients into clinically informative treatment categories would possibly benefit the 255,000 Americans and Canadians diagnosed with IDC each year [13, 14].

We previously performed global miRNA profiling using a Taqman Low Density Array (TLDA) for 71 lymph node-negative (LNN) IDC patient samples [9]. Herein, we report that further analyses of these data identified miR-449a to be highly overexpressed and significantly associated with increased incidence of patient relapse. Moreover, we revealed the role of miR-449a in increasing tumor progression *via* CRIP2 repression, highlighting the potential importance of this new pathway in driving aggressive cancer behavior both *in vitro* and *in vivo*.

## RESULTS

### Overexpression of miR-449a was associated with tumor recurrence in LNN breast cancer

**Figure 1:**
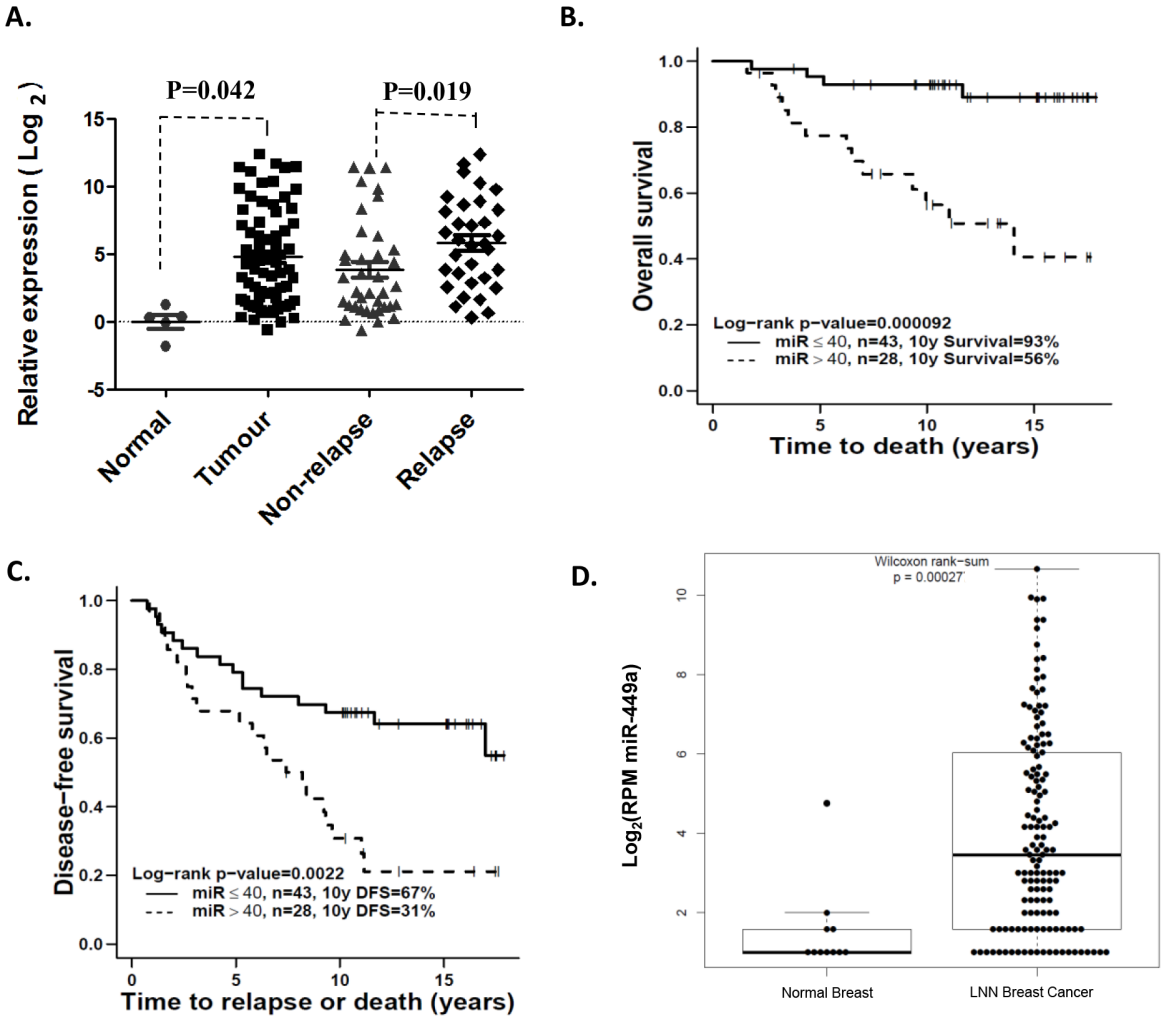
Overexpression of miR-449a was associated with higher risk of recurrence in lymph node-negative (LNN) breast cancer patients **A.** Taqman Low Density Array (TLDA) was used to identify that miR-449a was significantly increased in primary LNN breast cancer samples and strongly associated with tumor relapse. **B & C.** Kaplan-Meier survival curves showed a significant difference in both overall survival and disease-free survival when comparing high *vs.* low expression groups, segregated based on the expression value cut-off of 40 fold-change relative to the median expression of normal tissue samples. **D.** miRNA-Seq and clinical data from The Cancer Genome Atlas (TCGA) validated that miR-449a expression was significantly higher in LNN breast cancer samples (n=266) compared to adjacent normal tissue (n=87).

Taqman Low Density Array (TLDA) miRNA profiling demonstrated that miR-449a expression was significantly increased in pre-treatment primary LNN invasive ductal breast cancer samples (n=71) relative to normal mammary epithelial tissues (n=5) (p=0.042; Figure 1A). Additionally, miR-449a expression was significantly higher in the primary tumors of patients who eventually relapsed, when compared to the primary tumors of non-relapsed patients (p=0.019; Figure 1A).

In order to investigate the association between miR-449a expression and patient survival, the patient cohort was first segregated using the median miR-449a expression value (19.31 fold-change relative to the median expression of normal tissue samples) into high (n=36) *vs.* low (n=35) miR-449a expression groups. Using ten-year (minimum) clinical follow-up data, the high expression group was observed to have significantly worse overall survival (OS) (p=0.0013) relative to the low expression group. There was however, no significant difference in disease-free survival (DFS) between the high *vs.* low miR-449a expression groups (p=0.10) (Supplementary Figure S1A, S1B). We then utilized a second method for segregating the patient cohort to further investigate these clinical associations. Through an iterative statistical evaluation of all possible cut-off points, a segregation value of 40 fold-change relative to the median expression of normal tissue samples was chosen to dichotomize the 71 breast cancer patients into miR-449a high (n=28) *vs.* low (n=43) expression groups. Strikingly, the miR-449a high expression group experienced significantly poorer outcome in both OS and DFS (OS p=0.00092; DFS p=0.0022; Figure 1B, 1C). Together, these data indicated that breast cancer patients with higher miR-449a expression levels experienced higher recurrence rates and poorer clinical outcomes.

In TCGA data, the expression values for miR-449a were significantly higher in LNN breast cancer samples (n=266) compared to adjacent normal tissue samples (n=87) (Wilcoxon rank-sum test, p=0.00027; Figure 1D), validating our own observation of significant miR-449a overexpression in malignant LNN breast cancer tissue.

### Suppression of miR-449a significantly reduced *in vitro* cell survival, clonogenicity, migration, and invasion

**Figure 2:**
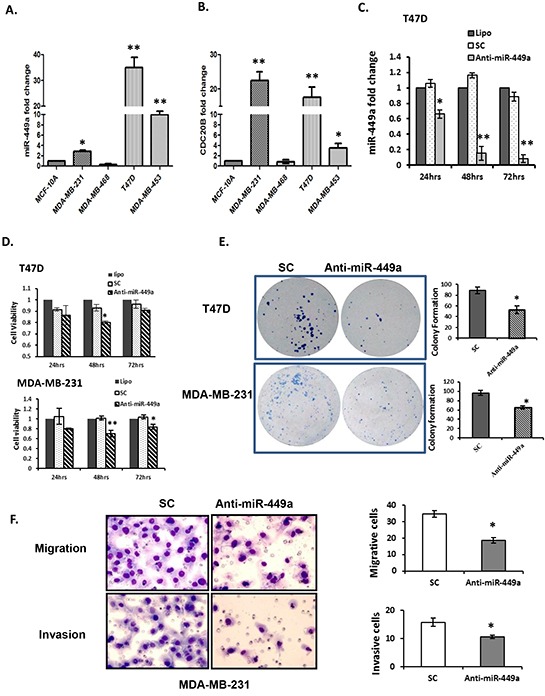
Downregulation of miR-449a reduced cell proliferation, clonogenicity, migration, and invasion in breast cancer cells **A.** Quantitative real-time PCR (qRT-PCR) for miR-449a expression in human normal mammary gland epithelial (MCF-10A) and breast cancer (MCF-10A, MDA-MB-231, T47D, and MDA-MB-453) cell lines. **B.** qRT-PCR for CDC20B, the miR-449a host gene, in corresponding cell lines. **C.** Anti-miR-449a (40 nM) was transfected into T47D cells and significantly reduced miR-449a expression compared with scrambled control (SC; 40 nM) or transfection reagent alone (Lipo). **D.** Cell viability was assessed in both T47D and MDA-MB-231 cells by the MTS assay 24–72 hours post-transfection. **E.** Clonogenic survival of T47D and MDA-MB-231 cells were assessed 14 days after transfection; representative images (*left*) and quantification (*right*). **F.** Representative images (*left*) and quantification (*right*) for the migration and invasion of MDA-MB-231 cells after transfection with anti-miR-449a or SC (40 nM). Data are presented as mean ± SEM; n=3; *p<0.05; **p<0.01.

The biological significance of miR-449a was evaluated in various human breast cancer cell lines. MiR-449a was significantly overexpressed in three (MDA-MB-231, T47D and MDA-MB-453) of the four cancer lines examined, when compared to normal mammary epithelial cells (MCF-10A; Figure 2A). Further, miR-449a is located within intron 2 of Cell Division Cycle 20B (*CDC20B*; Chromosome 5 - NC_000005.10), a cell cycle regulatory protein involved in anaphase nuclear movement and chromosome separation. As expected, quantitative real-time PCR (qRT-PCR) revealed that CDC20B was concordantly overexpressed in the three miR-449a overexpressing cancer lines (Figure 2B).

To evaluate the effects of miR-449a suppression, cells were transfected with either SC (negative control) or anti-miR-449a (Figure 2C). MiR-449a depletion significantly decreased cell viability in both T47D and MDA-M-231 cells, by 20% and 22% respectively, at 48 hours post-transfection (Figure 2D). Additionally, colony formation assays demonstrated that depletion of miR-449a significantly reduced clonogenicity of both T47D and MDA-MB-231 cells (Figure 2E).

Given the strong correlation of miR-449a overexpression with patient relapse, we investigated changes in cell migration and invasion after suppressing miR-449a. Because T47D cells do not migrate or invade, MDA-MB-231 cells were used for these experiments. Anti-miR-449a significantly reduced migration in MDA-MB-231 cells (19% *vs.* 36% for SC) (Figure 2F *top panel*). Furthermore, it reduced the invasive ability of the aggressive MDA-MB-231 cells to 10% *vs.* 16% for SC transfected cells (Figure 2F *bottom panel*).

### MiR-449a targeted the 3′-untranslated region (3′-UTR) of CRIP2

**Figure 3:**
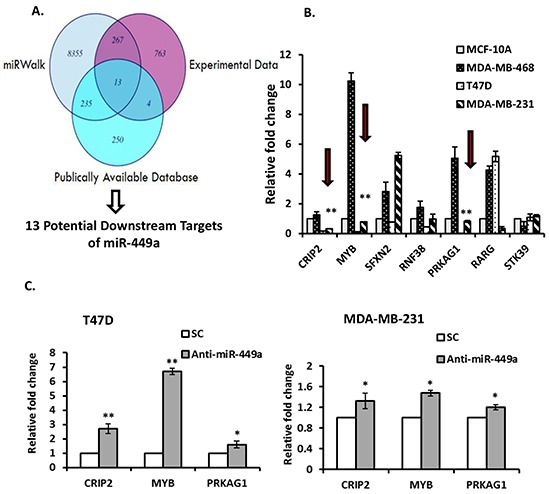
MiR-449a putative gene target identification **A.** Venn diagram illustrating the approach used to identify potential targets of miR-449a. *In silico* (miRWalk) data was combined with experimental (GeneChip Human Genome U133 Plus 2.0 mRNA array of T47D cells transfected with anti-miR-449a) and publically available data (lymph node negative (LNN) cancer gene profiling). **B.** The basal expression for the subset of genes that were potentially cancer-related was determined by quantitative real-time PCR (qRT-PCR). Consistently down-regulated genes in at least two cell lines are indicated (*red arrows*). **C.** T47D and MDA-MB-231 cells were transfected with anti-miR-449a (40 nM) or scrambled control (SC), and candidate miR-449a target gene expression was assessed using qRT-PCR 48 hours after transfection. Data are presented as mean ± SEM; n=3; *p<0.05; **p<0.01.

A three-pronged approach was undertaken to identify potential miR-449a targets as previously described [19]; miRWalk, experimental (GeneChip Human Genome U133 Plus 2.0 mRNA array of T47D cells transfected with anti-miR-449a), and publically available data [20] were all utilized (Figure 3A). Thirteen potential downstream targets were identified. Subsequently, Kyoto Encyclopedia of Genes and Genomes (KEGG) Pathway and Gene Ontology (GO) term enrichment analyses were performed to identify genes involved in essential cellular processes and/or tumor progression, such as cell cycle, apoptosis, DNA replication, and vascular neogenesis. Seven relevant genes were identified for validation: CRIP2, MYB, SFXN2, RNF38, PRKAG1, RARG and STK39. The baseline expression of each of these seven genes was examined by individual qRT-PCR. CRIP2, MYB and PRKAG1 were consistently under-expressed in both T47D and MDA-MB-231 cells (both of which have high endogenous expression of miR-449a), and overexpressed in MDA-MB-468 cells (which have low endogenous expression of miR-449a) relative to MCF-10A normal mammary gland epithelial cells (Figure 3B). Suppressing miR-449a in T47D and MDA-MB-231 cells resulted in up-regulated expression of CRIP2, MYB and PRKAG1 mRNA transcript levels at 48 hours post-transfection (Figure 3C).

**Figure 4:**
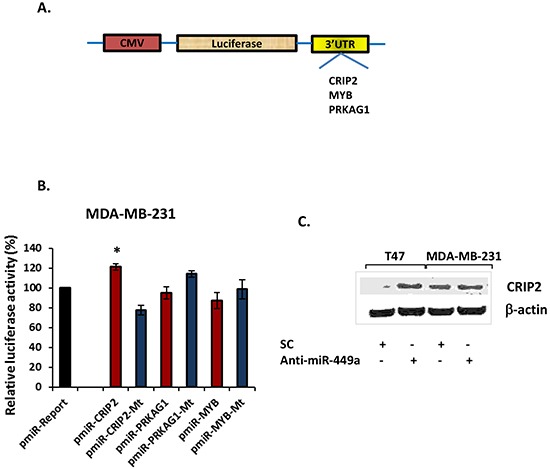
MiR-449a directly targeted the 3′-untranslated region (3′-UTR) of CRIP2 **A.** Schema depicting the luciferase reporter vectors carrying the predicted miR-449a binding sites downstream of the *Firefly luciferase* gene (pmiR-Report vector). **B.** Wild type or mutant reporter vector, with either scrambled control (SC; 40 nM) or anti-miR-449a (40 nM), were co-transfected into MDA-MB-231 cells, and luciferase activity was measured at 24 hours post-transfection. Vector and anti-miR-449a co-transfected luciferase activity was normalized to vector and SC co-transfected luciferase activity, with *Renilla* luciferase activity for transfection efficiency normalization. These data were then compared to the pmiR-Report control vector data. **C.** Western blot for CRIP2 in T47D and MDA-MB-231 cells, 48 hours after transfection. Data are presented as mean ± SEM; n=3; *p<0.05.

In order to determine whether miR-449a directly targets the 3′-UTRs of CRIP2, MYB, and/or PRKAG1, the 3′-UTR for each gene was cloned into separate pmiR-Report luciferase reporter vectors (Figure 4A). Cells were then co-transfected with anti-miR-449a and a luciferase reporter plasmid carrying the relevant wild type 3′-UTR sequences; luciferase activity was measured and normalized to the luciferase activity in cells co-transfected with SC and the same vector (*Renilla luciferase* was also transfected to normalize for transfection efficiency). In MDA-MB-231 cells (which endogenously express high levels of miR-449a; Figure 2A), CRIP2 3′-UTR luciferase (pmiR-CRIP2) expression was significantly higher in the presence of anti-miR-449a (Figure 4B). Moreover, when the CRIP2 3′-UTR was mutated (pmiR-CRIP2-Mt), the increase in luciferase activity was abrogated. Concordantly, in MDA-MB-468 cells (which endogenously under-express miR-449a; Figure 2A), CRIP2 3′-UTR luciferase expression was downregulated in the presence of pre-miR-449a (Supplementary Figure S1C). This downregulation was abrogated in the case of mutant CRIP2 3′-UTR luciferase vectors. MYB and PRKAG1 did not interact in a straight-forward manner with miR-449a (Figure 4, Supplementary Figure S1C), so were not investigated any further.

Western blotting confirmed increased protein expression for CRIP2 in both T47D and MDA-MB-231 cells in comparison to SC (Figure 4C). Together, the luciferase and Western blotting data support the specific and direct inhibition of CRIP2 by miR-449a.

### Stable expression of CRIP2 reduced *in vitro* cell viability, migration, and invasion, as well as *in vivo* tumorigenicity

CRIP2 has been reported to be a tumor-suppressor; its down-regulation was observed in esophageal squamous cell carcinoma and re-expression induced cell death [21]. To examine the biological significance of CRIP2 in breast cancer progression, we first evaluated endogenous CRIP2 expression in breast cancer cell lines. CRIP2 expression was significantly reduced in all tested cells in comparison to the normal control (MCF-10A; Supplementary Figure S2A).

**Figure 5:**
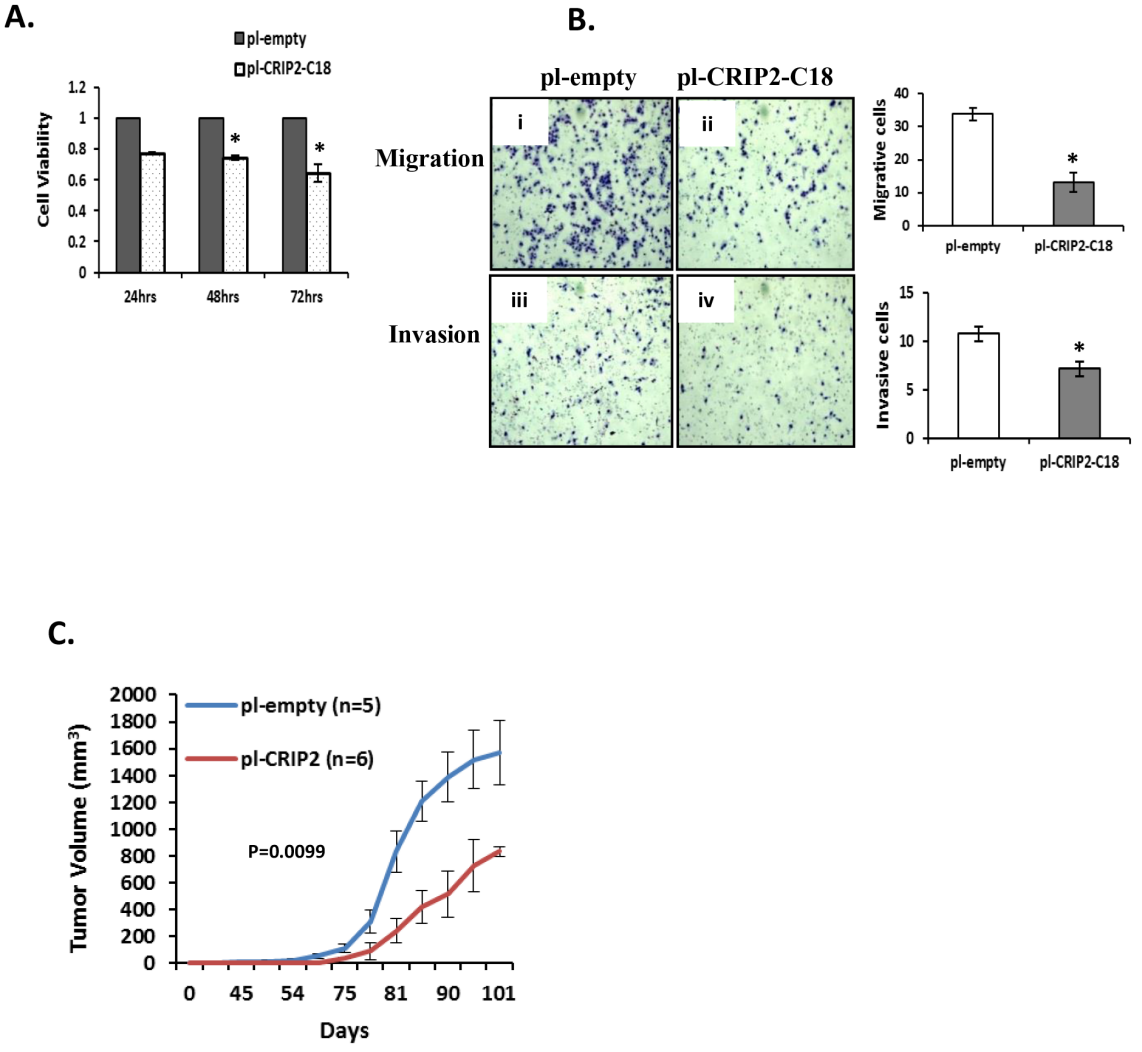
Stable overexpression of CRIP2 reduced tumorigenicity *in vitro* and *in vivo* **A.** Cell viability (MTS) was measured in MDA-MB-231 cells stably transfected with CRIP2 (pI-CRIP2-C18) or control vector (pI-empty). **B.** Representative images for migration and invasion of stably transfected MDA-MB-231 cells. **C.** Quantification of tumor volume. Data were presented as mean ± SEM; n=3 for *in vitro* assays; n=5 or 6 mice/group for *in vivo* assays; *p<0.05.

Next, a CRIP2 expression plasmid was transfected into MDA-MB-231 cells, and positive clones that stably expressed CRIP2 (C18 and C19) were identified by Western blot and qRT-PCR (Supplementary Figure S2B, S2C). All subsequent experiments were then conducted using the positive C18 clone (pI-CRIP2-C18), which expressed higher levels of CRIP2 than the C19 clone. The C18 clone demonstrated a significant reduction in cell viability compared to parental control cells (pI-empty), a trend that was observed for up to 72 hours (Figure 5A). Since tumor metastases are strongly associated with cell migration and invasion, these properties were assessed in the C18 clone *vs.* the empty control cells. As shown in Figure 5B, C18 cells exhibited a significant reduction in both migration and invasion potential compared to control cells. Furthermore, *in vivo* mouse experiments revealed significant suppression of tumor growth relative to empty control groups (n=6, p=0.0099; Figure 5C). Thus, both *in vitro* and *in vivo* data support a tumor suppressive activity of CRIP2 in breast cancer models.

### CRIP2 inhibited tumor angiogenesis

**Figure 6:**
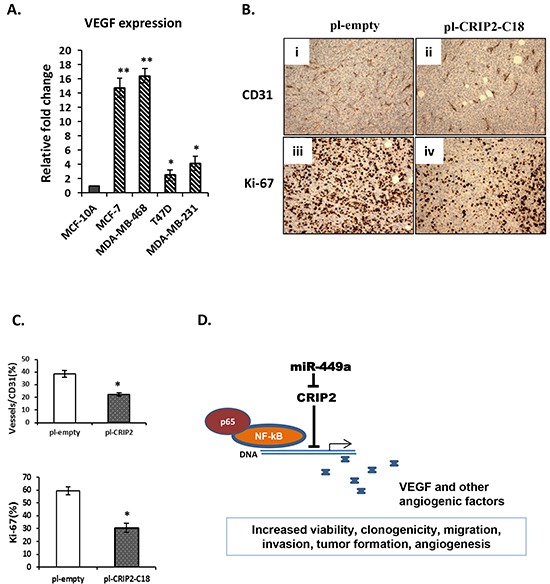
A proposed model for the miR-449a-CRIP2 pathway **A.** Quantitative real-time PCR (qRT-PCR) was used to assess VEGF expression in human normal mammary gland epithelial (MCF-10A) and breast cancer (MCF-10A, MDA-MB-231, T47D, and MDA-MB-453) cell lines. **B.** Microvascular density and cell proliferation were assessed using CD31 and Ki-67 immunohistochemistry, respectively, in mouse xenograft tumors formed by empty control (pI-empty) or CRIP-2 stably transfected cells (pI-CRIP2-C18). **C.** Quantification of CD31 and Ki-67 expression by counting cells in 5 randomly selected areas. The data are presented as mean ± SEM; n=3; *p<0.05; **p<0.01. **D.** Schema for the miR-449a-CRIP2 pathway. MiR-449a inhibits CRIP2, allowing the NF-kB/p65 complex to transcriptionally activate angiogenic factors such as VEGF.

A previous study in nasopharyngeal carcinoma demonstrated that CRIP2 overexpression in cell lines transcriptionally and functionally down-regulated NF-κB–mediated proangiogenic proteins such as IL-6, IL-8 and VEGF [22]. Thus, we assessed endogenous VEGF transcript expression in these breast cancer cell lines. There was indeed a significantly higher expression of VEGF in all breast cancer cell lines tested (Figure 6A) compared to the normal MCF-10A cells, similar to the lower levels of CRIP2 in the breast cancer cells compared to MCF-10A (Supplementary Figure S2), as well as our previously reported VEGF protein expression in archival human breast cancer samples [9].

CRIP2 has been implicated in VEGF-mediated blood vessel formation in mice [23]. To assess CRIP2-induced blood vessel formation in breast tumors, C18 tumors were established in mice, and compared to empty control tumors with respect to CD31 immunohistochemistry, a biomarker for blood vessel endothelium. CRIP2 expression significantly reduced tumor microvasculature density as well as decreased Ki-67 (a proliferative marker) expression (Figure 6B, 6C).

In summary, our findings support the model wherein overexpression of miR-449a in breast cancer suppresses CRIP2 activity, leading in turn to increased tumor growth, migration, invasion, as well as angiogenic signaling (Figure 6D).

## DISCUSSION

The biological complexities of breast cancer are a significant challenge in translational medicine. For patients with estrogen receptor-positive and LNN early stage breast cancer, gene-expression profiling (e.g. Oncotype DX) is an accepted clinical strategy for assessing risk recurrence and in the selection for adjuvant chemotherapy treatments [24, 25]. However, these approaches often have significant limitations, including lack of patient reported outcomes and no true comparative reference standards. In particular, the extent of clinical benefit with Oncotype DX remains to be documented [26, 27]. Given the recent advances in high-throughput sequencing technology, which revealed that more than 70% of the human genome can be transcribed, including thousands of non-coding RNAs [28, 29], it behooves us to examine these RNA species, particularly the microRNA family, which is well established as master regulators of tumor development and progression [9, 18, 30].

In this early-stage study, miR-449a was observed to be significantly up-regulated in a cohort of 71 primary LNN breast tumors, and its overexpression was associated with higher risk of recurrence and lower overall survival. MiR-449a overexpression promoted tumor cell proliferation and colony formation in two cell lines, also increasing migration and invasion. These effects were at least partially mediated *via* CRIP2 inhibition, which in turn also enhanced *in vivo* tumor formation and angiogenesis.

The role of miR-449a and miR-449b (another member of the miR-449 family cluster and also located in the second intron of *CDC20b*) in other human malignancies appears to be controversial, and varies largely depending on the biological context. Both miR-449a and miR-449b were first described as tumor suppressors in osteosarcoma cells, targeting *CDK6* and *CDC25A* [31]. Under-expression of these miRNAs has been reported in human prostate cancer tissue, resulting in the repression of HDAC-1 (histone deacetylase 1) [32]. Furthermore, miR-449a downregulation in non-small cell lung cancer correlated with the presence of lymph node metastasis, poor survival, and c-MET repression [33]. Growth arrest induced by miR-449a may also be dependent on Rb inhibition [34].

In contrast, several studies supported the oncogenic phenotype of miR-449a and miR-449b. MiR-449a overexpression was significantly increased in colorectal carcinoma and inversely correlated with the levels of serum carcinoembryonic antigen (CEA) [35]. High-throughput small RNA sequencing in peritoneal endometriotic lesions with matched healthy surrounding tissue indicated that miR-449a was one of five miRNAs expressed at significantly higher levels in the epithelial cells of endometriotic lesions [36]. In prostate cancer, microRNA profiling identified 31 microRNAs, including miR-449b, that were associated with recurrence [37]. In an independent cohort of 163 radical prostatectomy patients, miR-449b overexpression was shown to be an independent predictor of recurrence after prostatectomy [37].

The precise mechanism by which miR-449a is dysregulated remains unclear. However, we speculate that the function of miR-449a is fundamentally linked with cytogenetic location and is dependent on cancer type or tissue of origin. MiR-449a is located on chromosome 5 (5q11.2; intron 2 of *CDC20b*). This chromosomal location was identified as a highly susceptible region (5q11.2 SNP (rs889312)) in breast cancer (MIM 114480) for women of European ancestry, in early genome-wide association studies (GWASs) [38, 39].

Our study identified a novel target for miR-449a: Cysteine-Rich Protein 2 (CRIP2). CRIP2 belongs to the second class of LIM domain proteins, which usually lack DNA-binding homeodomains [40]. It is located on 14q32.3, a region frequently associated with chromosomal loss, and has been identified as an important tumor suppressor [22, 41]. In particular, nasopharyngeal carcinoma cell lines and tumors have decreased CRIP2. Re-expression of CRIP2 in these cells suppressed the transcription of NF-κB–mediated proangiogenic cytokines, such as IL-6, IL-8, and VEGF [22]. Although the cell line correlation between endogenous miR-449a (Figure 2A), CRIP2 (Supplementary Figure S2A), and VEGF (Figure 6) expression did not correlate precisely, differences would be expected due to the concentrations of alternate endogenous miRNA targets and competing endogenous RNA cross-talking [42]. Nonetheless, in the current study, we have successfully demonstrated a direct expression and functional changes in these relationships both *in vitro* and *in vivo* for breast cancer.

In summary, we have identified the potential clinical relevance of miR-449a in LNN breast cancer, wherein it promotes a variety of oncogenic functions such as increased cell viability, colony formation, migration, and invasion. MiR-449a suppresses CRIP2 expression, which then leads to increased tumor formation (as well as migration and invasion), along with the activation of proangiogenic cytokines such as VEGF, possibly *via* the NF-κB/p65 complex. Taken together, our study highlights the importance of this novel pathway in driving aggressive breast cancer behaviour, and identifies potential therapeutic opportunities.

## MATERIALS AND METHODS

### Patient samples

As part of a previous study, 71 formalin-fixed and paraffin-embedded (FFPE), early stage, pre-treatment LNN IDC samples were collected from a Phase III clinical trial of patients treated with Tamoxifen alone or in combination with local breast radiotherapy [9, 10]. Global miRNA expression profiling using the TLDA platform was conducted on these samples, and previously published [9]. These studies have all been approved by the University Health Network Research Ethics Board.

All of The Cancer Genome Atlas (TCGA)'s available normalized miRNA-Seq and clinical data for breast cancer were downloaded from the Broad Firehose (stddata run 2015_04_02, n = 737) [15].

### Cell lines and transfection

Human breast cancer cell lines MDA-MB-231 (derived from pleural effusion), MDA-MB-468 (derived from pleural effusion), MDA-MB-453 (derived from pericardial effusion), and T47D (derived from pleural effusion) were obtained from American Type Culture Collection (ATCC), and cultured in either α-MEM or RPMI 1640 supplemented with 10% fetal bovine serum (FBS). Human normal mammary gland epithelial cells (MCF-10A) were obtained from Dr. Linda Penn (Princess Margaret Cancer Centre Research Institute) and cultured in DMEM/HAM F12 supplemented with 5% horse serum, insulin, hEGF, hydrocortisone (Clonetics), and cholera toxin (Sigma-Aldrich). All cell lines were maintained at 37°C and 5% CO2, authenticated using Short Tandem Repeat analyses, and tested to be free from mycoplasma contamination.

Anti-miR-449a or pre-miR-449a mimic (Ambion) were reverse transfected into cells using Lipofectamine 2000 (Invitrogen) at a final concentration of 40 nM (unless otherwise indicated), according to the manufacturer's instructions.

CRIP2-expressing vectors were purchased (Applied Biological Materials) and transfected into MDA-MB-231 cells using Lipofectamine 2000. Independent single clones were obtained after 2 weeks of drug selection. qRT-PCR and Western blot were used to screen for positive clones.

### Quantitative real-time PCR (qRT-PCR) for mRNA and miRNA Expression

Total RNA was isolated using the RecoverAll Total Nucleic Acid Isolation kit (Ambion) for FFPE samples, or the Total RNA purification kit (Norgen BioTek) for cell lines. For mRNAs, reverse transcription was performed using SuperScript III Reverse Transcriptase (Invitrogen) according to the manufacturer's instructions. qRT-PCR analyses were performed using SYBR Green (ThermoFisher) and the ABI PRISM 7900 Sequence Detection System (Applied Biosystems). The primer sequences used in this study are listed in Supplementary Table S1. The relative fold change in RNA expression was calculated using the 2-ΔΔCt method [16], where the average of ΔCt values for the amplicon of interest was normalized to that of an endogenous gene (GAPDH), compared with control specimens.

MicroRNA expression was evaluated using Taqman MicroRNA Assays (Applied Biosystems). RNA was reverse-transcribed with a MultiScribe reverse transcriptase using a stem-loop RT primer designed to specifically hybridize with an individual miRNA. The RT products were subsequently amplified with sequence-specific primers using the Applied Biosystems 7900 HT Real-Time PCR system. RNU48 was used as an endogenous control.

### Viability, clonogenic, migration, and invasion assays

The soluble tetrazolium salt (MTS; Promega) assay was used to assess cell viability at 24, 48, and 72 hours post-transfection according to the manufacturer's specifications. The clonogenic assay was performed on MDA-MB-231 and T47D cells as previously described [17]. Briefly, cells were transfected in 12-well plates. After 48 hours, cells were harvested and re-seeded onto 6-well plates in triplicate. After 14 days' incubation, the plates were fixed and stained, and the number of colonies counted. The fraction of surviving cells was calculated by comparing with cells treated with scramble control (SC).

Cell migration and invasion assays were performed using BD Control Inserts and BioCoat Matrigel Invasion Chambers (BD Biosciences). Transfected cells were seeded on either control inserts (polyethylene terephthalate membrane) or trans-well chambers with Matrigel. Briefly, RPMI (2 mL) supplemented with 15% FBS was added to the lower chamber, serving as the chemo-attractant. Transfected cells were then re-suspended in RPMI with 1% FBS, and added to the upper chamber (0.5 mL). Twenty hours later, migrating or invading cells attached to the lower surface of the membrane insert were fixed, stained, and counted under a microscope. Relative migration was calculated relative to cells transfected with SC. The percentage invasion was calculated based on the number of cells that invaded through the matrigel insert, divided by the number of cells that migrated through the control insert.

### Luciferase reporter assay

The 3′-untranslated regions (3′-UTRs) of potential RNAs targeted by miR-449a were amplified by PCR and placed in the pmiR-Report vector (Ambion), downstream of the *Firefly luciferase* gene. Empty and mutant sequences were used in control plasmids. These vectors (pmiR) were co-transfected with pre-miR-449a, anti-miR-449a, or SC into various cell lines. pRL-SV vector (Promega), containing *Renilla luciferase*, was also co-transfected with each condition as a reference control. At 48 hours post-transfection, Firefly and Renilla luciferase activities were measured using the Dual-Luciferase Reporter Assay (Promega).

### Western blotting

Anti-miR-449a or SC transfected cells were collected and lysed at 72 hours. Total protein was extracted and quantified using the BCA method. SDS-PAGE (15%) was performed with 20 μg of protein per well, and samples were transferred onto PVDF membranes (Millipore). Proteins were detected using CRIP2 (1:1200; Abcam), or PRKAG1 (1:1000; Abcam) antibodies. Signals were visualized using the ECL Western Blotting Substrate kit (Pierce).

### *In vivo* tumorigenicity and immunohistochemistry

All mouse experiments were conducted in compliance with the University Health Network's Animal Resource Centre guidelines. Cell line tumorigenicity was assayed by subcutaneous (s.c.) injection. As described previously [18], 1×10^7^ cells were mixed with 100 μL of matrigel and injected into 6- to 8-week-old female severe combined immunodeficient (SCID) mice. Tumor volume was measured twice weekly using a caliper, and calculated as tumor length × width2/2. Tumor tissues were collected and fixed with 10% buffered formalin. Immunohistochemistry analyses were performed on 4 μm FFPE tumor sections using the Ventana Autostainer (BenchMark XT; Ventana Medical Systems). Rabbit polyclonal CD31 (sc-1506-R, 1/1000 dilution; Santa Cruz), or mouse Ki-67 antibody (clone MIB1, M7240, 1:100 dilution; Dako) were used.

### Statistical analyses

Survival curves were generated using the Kaplan-Meier method and compared using the log-rank test. All experiments were performed at least three times independently unless otherwise stated. Data are presented as mean + standard error of the mean (SEM). A p-value of less than 0.05 was considered to be statistically significant.

## SUPPLEMENTARY FIGURES AND TABLE


