# Supplementary material for: MiR-449a promotes breast cancer progression by targeting CRIP2
Source: Oncotarget. 2016 Feb 26;7(14):18906–18. doi: 10.18632/oncotarget.7753 (PMC4951339; doi:10.18632/oncotarget.7753)
Supplement: Supplementary file 1 [file oncotarget-07-18906-s001.pdf]

SUPPLEMENTARY FIGURES AND TABLE

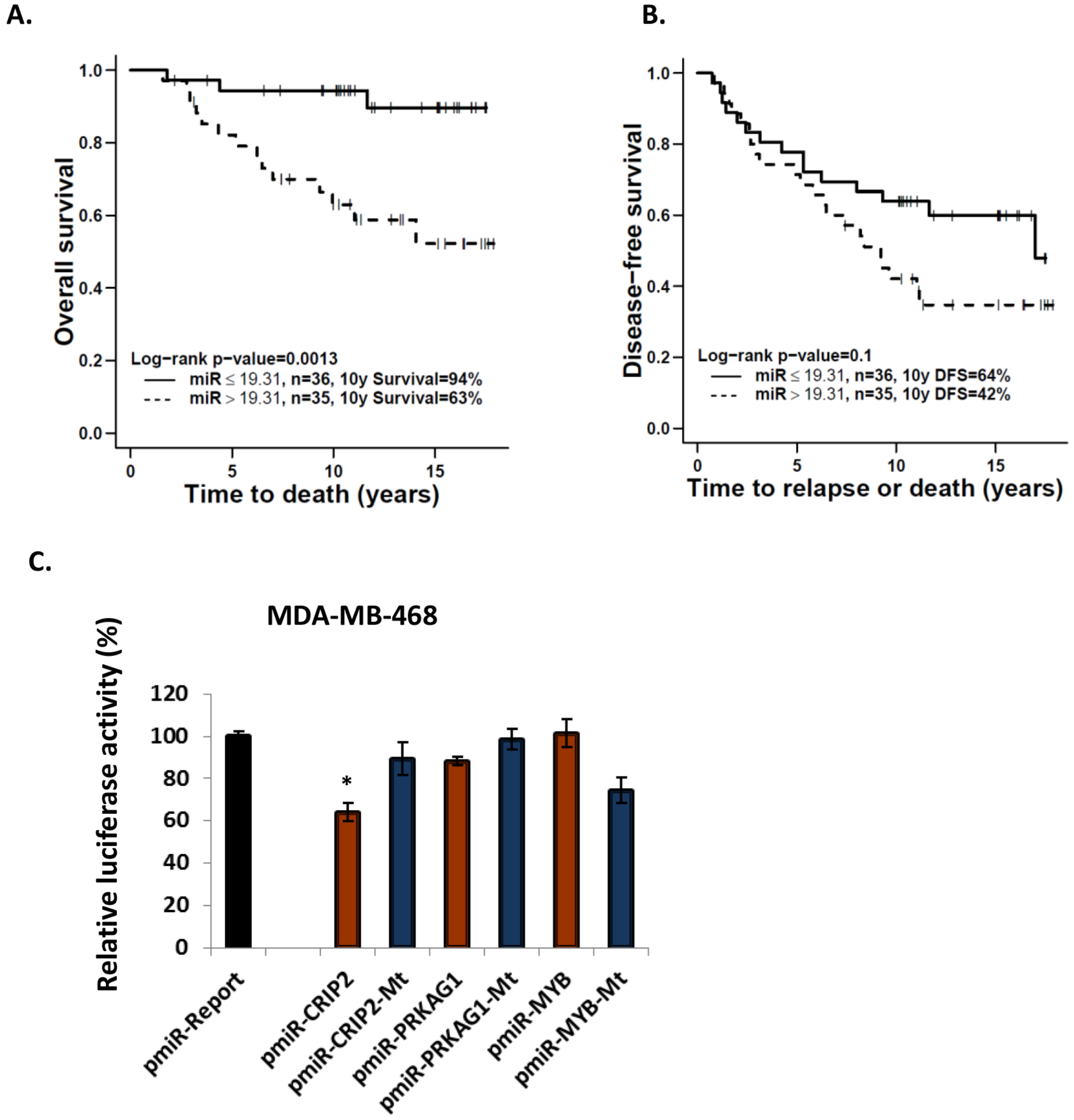

Supplementary Figure S1: MiR-449a Supplemental Data. A. Kaplan-Meier plot for overall survival (OS) as a function of miR-449a expression. The median miR-449a expression value (19.31 fold-change relative to the median normal tissue expression) was used to separate high (n=36) vs. low (n=35) groups. **B.** Kaplan-Meier plot for disease-free survival (DFS) as a function of miR-449a expression. **C.** Wild type or mutant reporter vector, with either scrambled control (SC; 40 nM) or pre-miR-449a (40 nM), were co-transfected into MDA-MB-468 cells, and luciferase activity was measured at 48 hours post-transfection. Vector and pre-miR-449a co-transfected luciferase activity was normalized to vector and SC co-transfected luciferase activity, with Renilla luciferase activity for transfection efficiency normalization. These data were then compared to the pmiR-Report control vector data. Data are presented as mean ± SEM; n=3; *$p<0.05$.

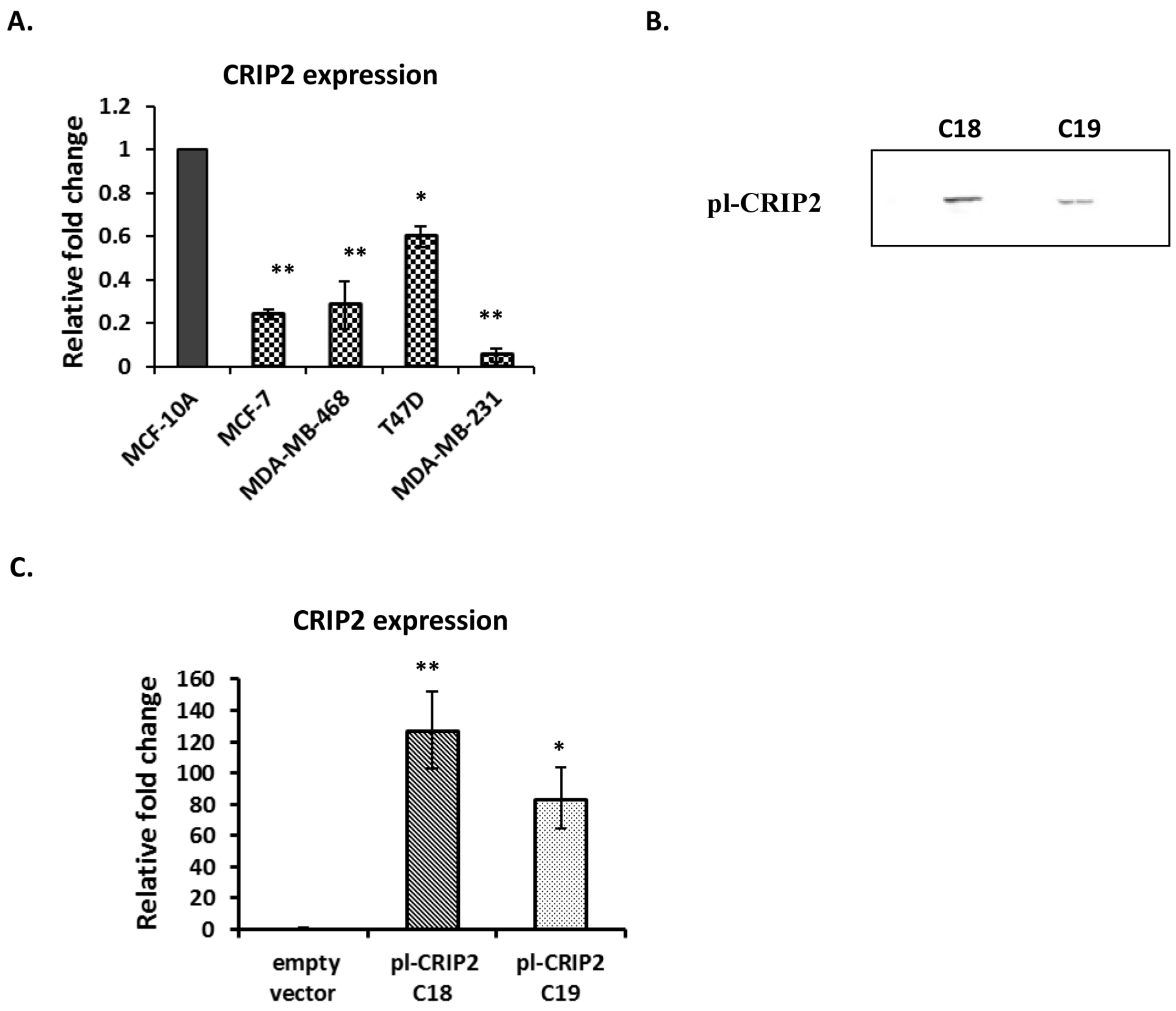

Supplementary Figure S2: CRIP2 Supplemental Data. A. Quantitative real-time PCR (qRT-PCR) for CRIP2 expression in breast cell lines. **B.** CRIP2 overexpressing clones (C18, C19) confirmed by Western blot. **C.** qRT-PCR confirming high levels of CRIP2 expression in two selected clones. Data are presented as mean ± SEM; n=3; *$p<0.05$; **$p<0.01$.

Supplementary Table S1: Primers used for Quantitative Real-Time PCR (qRT-PCR)

Primer	Forward (For) or Reverse (Rev) Primer	5' to 3' Primer Sequence
**CDC20B**	For	gaagacaccgcctgagaaag
	Rev	cacagagctgcatttttcca
**CRIP2**	For	actgatgcctcctcaccatc
	Rev	tgtttgtgagccaaccagag
**MYB**	For	ggcagaaatcgcaaagctac
	Rev	gcagggagttgagctgtagg
**SFXN2**	For	cagctgctgtatgccaagaa
	Rev	aaggactggttcacccactg
**RNF38**	For	ttaaacctggcagagcgact
	Rev	caacacacttggcatggaac
**PRKAG1**	For	gtccctgcaggtgaagaaag
	Rev	gcagacaagcggtttaaagg
**RARG**	For	tcaaagctgcctgcctagat
	Rev	gcaaaggcaaagacaaggtc
**STK39**	For	ctagcaacagggggtgatgt
	Rev	atttgtgataaggcgctgct
**VEGF**	For	aaggaggagggcagaatcat
	Rev	atctgcatggtgatgttgga
